# Neuroprotective and Anti-Inflammatory Effects of Evernic Acid in an MPTP-Induced Parkinson’s Disease Model

**DOI:** 10.3390/ijms22042098

**Published:** 2021-02-20

**Authors:** Seulah Lee, Yeon Ji Suh, Seonguk Yang, Dong Geun Hong, Akihito Ishigami, Hangun Kim, Jae-Seoun Hur, Seung-Cheol Chang, Jaewon Lee

**Affiliations:** 1 Department of Pharmacy, College of Pharmacy, Pusan National University, Busan 46241, Korea; leeseulah@pusan.ac.kr (S.L.); duswl9449@naver.com (Y.J.S.); ajy020603@gmail.com (S.Y.); dgni21@naver.com (D.G.H.); 2 Molecular Regulation of Aging, Tokyo Metropolitan Institute of Gerontology, Tokyo 173-0015, Japan; ishigami@tmig.or.jp; 3 College of Pharmacy, Sunchon National University, Suncheon 57922, Korea; hangunkim@scnu.ac.kr; 4 Korean Lichen Research Institute, Sunchon National University, Suncheon 57922, Korea; jshur1@scnu.ac.kr; 5 Department of Cogno-Mechatronics Engineering, College of Nanoscience and Nanotechnology, Pusan National University, Busan 46241, Korea; s.c.chang@pusan.ac.kr

**Keywords:** Parkinson’s disease, evernic acid, 1-methyl-4-phenyl-1,2,3,6-tetrahydropyridine, neuroprotection, neuroinflammation, anti-inflammation

## Abstract

Oxidative stress, mitochondrial dysfunction, and neuroinflammation are strongly associated with the pathogenesis of Parkinson’s disease (PD), which suggests that anti-oxidative and anti-inflammatory compounds might provide an alternative treatment for PD. Here, we evaluated the neuroprotective effects of evernic aid (EA), which was screened from a lichen library provided by the Korean Lichen Research Institute at Sunchon National University. EA is a secondary metabolite generated by lichens, including *Ramalina, Evernia,* and *Hypogymnia*, and several studies have described its anticancer, antifungal, and antimicrobial effects. However, the neuroprotective effects of EA have not been studied. We found that EA protected primary cultured neurons against 1-methyl-4-phenylpyridium (MPP^+^)-induced cell death, mitochondrial dysfunction, and oxidative stress, and effectively reduced MPP^+^-induced astroglial activation by inhibiting the NF-κB pathway. In vivo, EA ameliorated MPTP-induced motor dysfunction, dopaminergic neuronal loss, and neuroinflammation in the nigrostriatal pathway in C57BL/6 mice. Taken together, our findings demonstrate that EA has neuroprotective and anti-inflammatory effects in PD models and suggest that EA is a potential therapeutic candidate for PD.

## 1. Introduction

Parkinson’s disease (PD) is a common movement disorder characterized by bradykinesia, rigidity, and slow movements accompanied by nonmotor symptoms such as anxiety, sleep disorder, and dementia. The neurodegenerative features of PD include dopaminergic neuronal loss in the substantia nigra (SN), accumulation of α-synuclein, and chronic neuroinflammation [1]. Etiological factors responsible for PD are not fully understood as well as for other neurodegenerative diseases such as Alzheimer’s disease and amyotrophic lateral sclerosis. While present clinical treatments aim to recover dopamine levels or inhibit endogenous dopamine degradation, it was recently suggested treatment strategies might be directed toward removing direct causes of neurodegeneration by suppressing chronic neuroinflammation [2,3]. In addition, several studies have presented evidence that activated microglia and reactive astrocytes induce neurodegeneration [4,5], which suggests anti-inflammatory agents offer a potential means of targeting PD.

Lichens are interesting plant-like, composite, symbiotic organisms that arise from cohabitations between green algae or cyanobacteria in filamentous fungi, and ubiquitously reside on rock surfaces, tundra, and even deserts. To date, more than 20,000 species have been identified. Lichens produce several secondary metabolites, such as depside, depsidone, xanthone, and dibenzofuran, which have been used in foods, perfumes, and medicines, and as dyes [6,7,8]. Furthermore, the metabolites produced by lichens exhibit antioxidant, antimicrobial, anticancer, antiviral, and anti-inflammatory effects [9,10,11,12,13].

Oakmoss (*Evernia prunastri*) is a common lichen species, which as its name implies is found on the surfaces of oak trees and by hydrolyzing depsides is also responsible for the odor of oak. Oakmoss is commercially harvested at ~700 tons annually in south-central Europe for the French fragrance industry [14]. Evernic acid (EA) is a major component of *Evernia prunastri* and is also an abundant metabolite in *Hypogymnia physodes*, *Ophioparma ventosa, Ramalina* species, and others and is classified as a depside with two monoaromatic rings [8]. Studies have reported EA is biologically active and that it severely inhibits the growths of the pathogenic fungi *Pythium ultimum* and *Phytophthora infestans* [15]. Another study showed that EA extracted from *Ramalina fastigiata* has antioxidant capacity and an antimicrobial effect on *E. coli* [16]. Others reported EA inhibits the proliferations of U-87 human glioblastoma cells and HeLa cells (a cervical cancer cell line) [17,18]. However, only one study has reported that it has a protective effect on U373-MG glioblastoma and SH-SY5Y neuroblastoma cells [13], and to date, no report has been issued on its neuroprotective effects in the presence of the neurodegenerative disease.

In a previous study, we reported on the neuroprotective effects of usnic acid, which is the most common dibenzofuran derivative found in several lichen species (e.g., *Usnea, Cladonia, Hypotrachyna, Lecanora,* and *Evernia*). In this previous study, usnic acid effectively suppressed glial activation but did not protect primary neurons against MPP^+^, which indicated its neuroprotective mechanism in PD is mediated by suppressing neuroinflammation [19]. Other studies have also reported that usnic acid is highly cytotoxic to gastric, breast, and pancreatic cancer cells and primary neurons [12,20]. Here, we screened neuroprotective candidates using a lichen extract library, and after finding EA has non-cytotoxic and neuroprotective effects, we investigated whether it has therapeutic potential in PD using cellular models and a MPTP-induced murine model.

## 2. Results

### 2.1. Screening of EA as a Neuroprotective Candidate in the Lichen Extract Library

**Figure 1:**
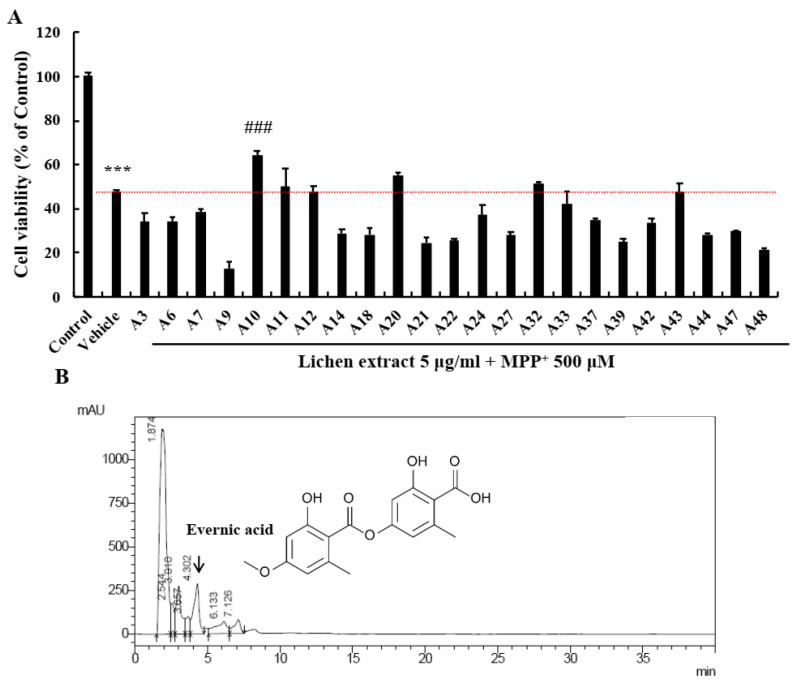
Screening for neuroprotective candidates in the lichen library. (**A**) Lichen extracts were tested to determine whether they were neuroprotective using an MTT assay. Results are presented as means ± standard errors (SEs) (*n* = 4). *** *p* < 0.001 vs. non-treated controls and ### *p* < 0.001 vs. MPP^+^-treated primary neurons (ANOVA with Fisher’s PLSD procedure). (**B**) HPLC data showed that the most abundant component in A10 was evernic acid.

The lichen extract library was provided by the Korean Lichen Research Institute (KoLRI) in Sunchon National University (Suncheon, Korea) and neuroprotective effects were screened using primary cultured neurons. We used acetone extracts isolated from 23 Korean lichen species representing 6 families. Primary neurons were sufficiently matured by culture for 1 week, pre-treated with lichen extracts at 5 μg/mL for 6 h, and then co-treated with MPP^+^ 500 μM for 24 h. MTT assays showed that MPP^+^ reduced cell viability by >50%. Of the 23 extracts examined, A10 most effectively prevented cell loss (Figure 1A). HPLC (high-performance liquid chromatography) was used to identify the component of A10 responsible. The most abundant component of A10 was EA (Figure 1B, black arrow), followed obtusatic acid and colensoic acid. Therefore, we supposed that EA was the component responsible for neuroprotective effect of A10. For uniformity and quality of results, next experiments were conducted using pure EA purchased from Santa Cruz Technology (Santa Cruz, CA, USA).

### 2.2. EA Protected Primary Neurons from MPP^+^-Induced Apoptosis

**Figure 2:**
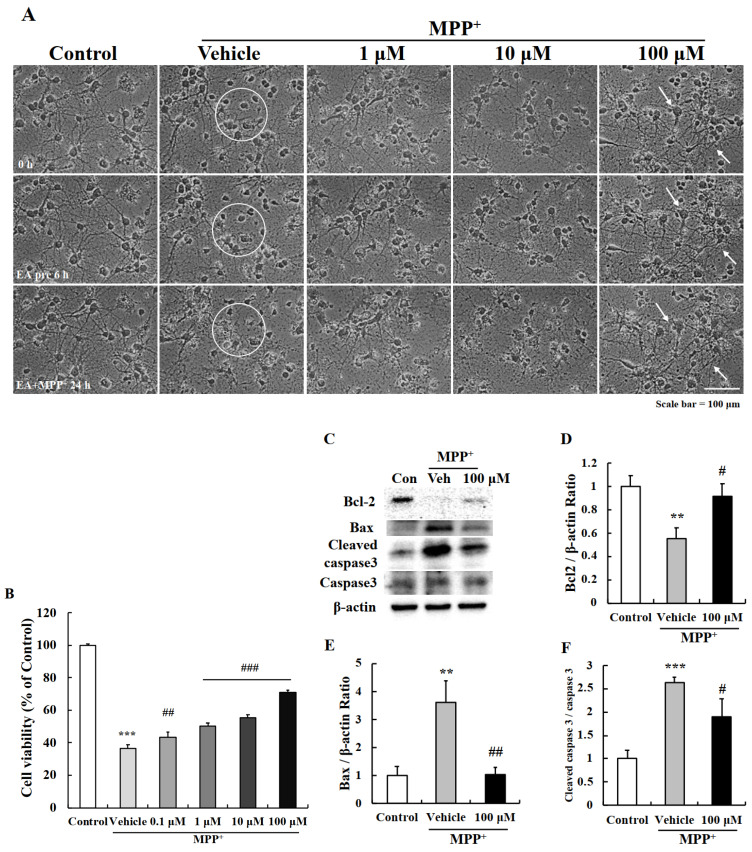
Anti-apoptotic effect of EA in primary neurons. (**A**) Representative images showed MPP^+^-induced neuronal cell death (white circle) and that pretreatment with 100 µM EA had a protective effect. Scale bar = 100 µm. (**B**) MTT assay confirmed that EA protected cells from MPP^+^-induced cell death. Values are means ± SEs (*n* = 8). *** *p* < 0.001 vs. naïve controls and ### *p* < 0.001 vs. MPP^+^-treated controls (the analysis was performed using ANOVA with Fisher’s PLSD procedure). (**C**) Western blot analysis confirmed that EA downregulated apoptotic markers in MPP^+^ treated primary neurons. (**D**–**F**) Bar graphs of fold changes of western blot. More than three independent experiments were performed and values are means ± SEs (*n* = 3–5). ** *p*, *** *p* < 0.01, 0.001 vs. naïve controls and # *p*, ## *p* < 0.05, 0.01 vs. MPP^+^-treated controls (the analysis was performed using ANOVA with Fisher’s PLSD procedure).

To confirm the neuroprotective effect of EA, primary cultured neurons were pre-treated with 1, 10, or 100 μM of EA for 6 h and then were co-treated with 500 μM of MPP^+^ for 24 h. Observations showed that treatment with MPP^+^ alone for 24 h shortened neurites and condensed cell bodies (white circle) and that EA pretreatment significantly reduced MPP^+^-induced neurite shortening (white arrows) (Figure 2A). In addition, MTT assays showed that EA pretreatment significantly and concentration-dependently protected MPP^+^-induced reductions in cell viability (Figure 2B). We next performed western blot analysis to check apoptotic factors since these observed MPP^+^-induced morphological changes are hallmarks of apoptosis [21]. MPP^+^ treatment reduced Bcl-2 (anti-apoptotic) and increased Bax (pro-apoptotic) protein levels, and 100 μM EA pretreatment prevented these changes (Figure 2C–F). These results demonstrated that EA suppressed MPP^+^-induced apoptosis in primary neurons.

### 2.3. EA Suppressed MPP^+^-Induced Mitochondrial Dysfunction in Primary Neurons

**Figure 3:**
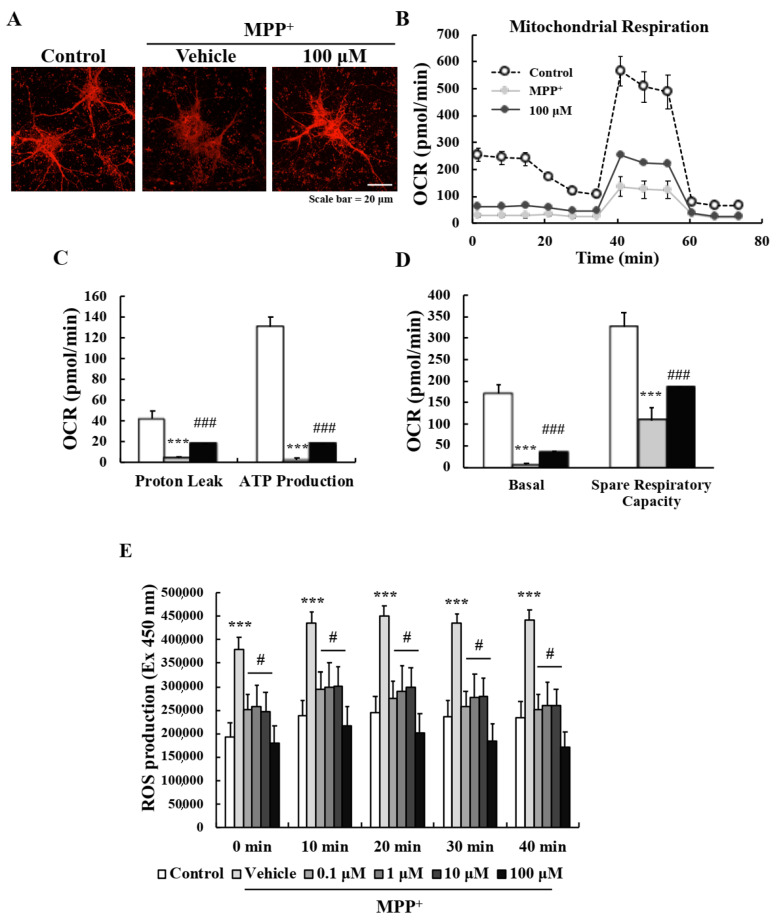
EA reduced MPP^+^-induced oxidative stress and mitochondrial dysfunction in primary neurons. (**A**) Representative images showing that 100 µM EA pretreatment enhanced MMPs. Scale bar = 20 µm. (**B**–**D**) Oxygen consumption rates of primary neurons were measured using the Seahorse Cell Mito Stress assay to assess mitochondrial respiratory functions after co-treatment with MPP^+^- and EA+MPP^+^. Two independent experiments were performed, and results were averaged. Values shown are means ± SEs (*n* = 4). *** *p* < 0.001 vs. naïve controls and ### *p* < 0.001 vs. MPP^+^ controls (the analysis was performed using ANOVA with Fisher’s PLSD procedure). (**E**) Intracellular ROS levels were measured using DCF-DA dye at 10 min intervals. Values are means ± SEs (*n* = 8). *** *p* < 0.001 vs. naïve controls and # *p* < 0.05 vs. MPP^+^ controls (the analysis was performed using ANOVA with Fisher’s PLSD procedure).

Mitochondrial dysfunction is the most well-known pathologic characteristic of PD. MPP^+^ induces a parkinsonian condition in vitro by blocking complex I of the electron transport chain (ETC) of mitochondria [22]. To investigate whether EA protects mitochondrial function, we measured mitochondrial membrane potentials (MMPs) and respiratory function. MitoTracker staining was used to detect active mitochondria, and MPP^+^ was found to reduce fluorescence intensities, whereas pretreatment with 100 μM EA suppressed these reductions (Figure 3A). In addition, the Seahorse XF Cell Mito Stress test sensitively showed that MPP^+^ diminished mitochondrial respiratory function by reducing proton leakage and ATP production, and that pretreatment with 100 μM EA significantly inhibited this MPP^+^-induced reduction (Figure 3B,D). ROS (reactive oxygen species) levels were measured using DCF-DA as abnormal mitochondria can induce excessive amounts [23,24]. As was expected, MPP^+^-induced ROS levels were significantly reduced by EA pretreatment (Figure 3E). These results demonstrate that the neuroprotective effects of EA involve the protection of mitochondrial function and antioxidant activity.

### 2.4. EA Inhibited MPP^+^-Induced Astrocyte Activation

**Figure 4:**
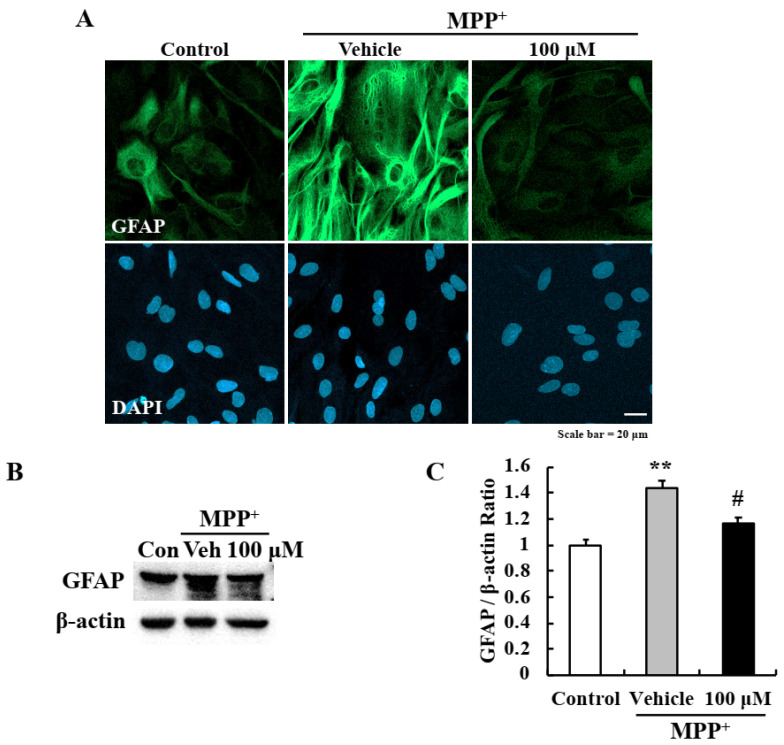
EA suppressed MPP^+^-induced glial activation in primary astrocytes. (**A**) Representative images showing that EA attenuated GFAP fluorescence intensity (an astrocyte marker); scale bar = 20 µm. (**B**) Western blot analysis confirmed that EA reduced glial activation by MPP^+^. (**C**) Densitometric western blot results. Three independent experiments were performed (*n* = 3). ** *p* < 0.01 vs. naïve controls and # *p* < 0.05 vs. MPP^+^ controls (the analysis was performed using ANOVA with Fisher’s PLSD procedure).

In a previous study, we found that although usnic acid did not directly protect primary neurons it suppressed astrocyte activation [19]. To determine whether EA also has a beneficial anti-inflammatory effect, primary astrocytes were pre-treated with EA for 6 h and then co-treated with MPP^+^ for 24 h. Pretreatment with 100 μM EA reduced MPP^+^-induced glial fibrillary acidic protein (GFAP) expression—a known astrocyte marker (Figure 4A), and this was confirmed by Western blotting (Figure 4B,C).

### 2.5. EA Inhibited the NF-κB Signaling Pathway in Primary Astrocytes

**Figure 5:**
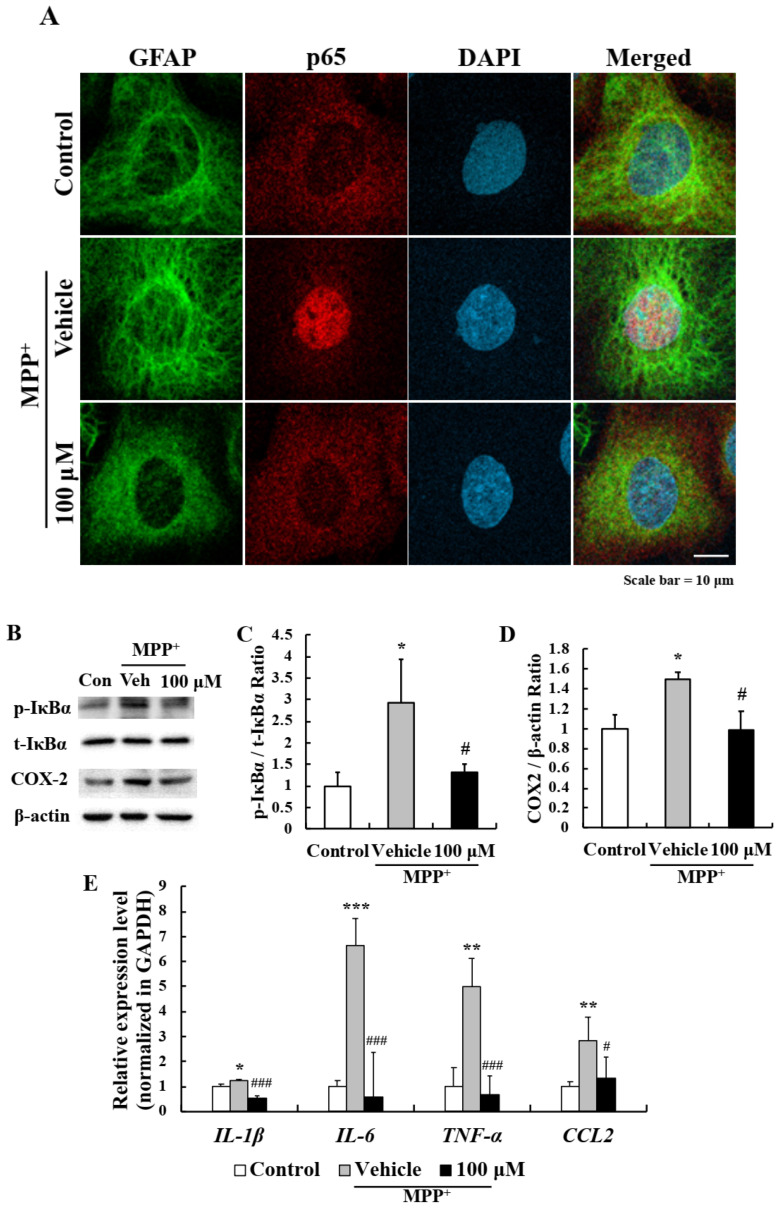
NF-κB pathway inhibition in primary astrocytes was responsible for the anti-inflammatory effect of EA. (**A**) Representative images showing that EA inhibited the MPP^+^-induced nuclear translocation of p65. Scale bar = 10 µm. (**B**) Western blot showed that EA repressed MPP^+^-induced IκBα phosphorylation and COX2 expression. (**C,D**) Bar graphs of fold changes of western blot. Three independent experiments were performed (*n* = 3). * *p* < 0.05 vs. naïve controls and # *p* < 0.05 vs. MPP^+^ controls (the analysis was performed using ANOVA with Fisher’s PLSD procedure). (**E**) Real-time PCR showed that EA significantly reduced the expressions of MPP^+^-induced inflammatory cytokines and chemokine. Values are means ± SEs (*n* = 3). * *p* < 0.05, ** *p* < 0.01, *** *p* < 0.001 vs. naïve controls and # *p* < 0.05, ### *p* < 0.001 vs. MPP^+^ controls (the analysis was performed using ANOVA with Fisher’s PLSD procedure).

To investigate the molecular mechanism responsible for the suppression of astrocyte activation by EA, primary astrocytes were pre-treated with EA for 6 h and then co-treated with MPP^+^ for 1 h. Interestingly, p65 monomer (a component of NF-κB) was translocated to nuclei when astrocytes were treated with MPP^+^ and this was inhibited by 100 μM EA pretreatment (Figure 5A). Western blot showed that MPP^+^ increased the phosphorylation of IκBα, which retains p50/p65 NF-κB dimer in cytoplasm, and that EA pretreatment also significantly reduced this effect (Figure 5B,C). Furthermore, EA reduced the MPP^+^-induced levels of COX-2 (an inflammatory factor downstream of NF-κB) (Figure 5B,D), and real time PCR showed that MPP^+^-induced upregulations of inflammatory cytokines (IL-1β, IL-6, and TNF-α) and CCL2 (a chemokine) were significantly suppressed by EA (Figure 5E). These results show that the anti-inflammatory effect of EA in primary astrocytes is due to blocking of the NF-κB signaling pathway.

### 2.6. EA Attenuated Motor Dysfunction in the MPTP-Induced PD Mouse Model

**Figure 6:**
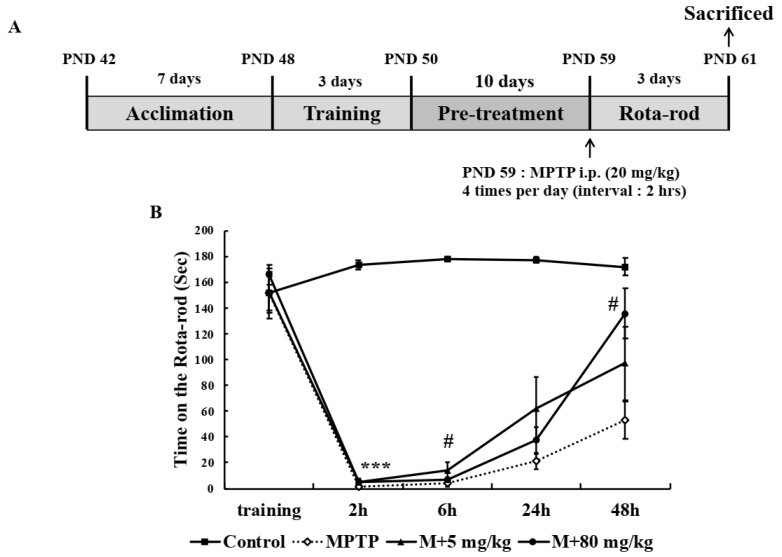
EA enhanced MPTP-induced motor dysfunction in mice. (**A**) Schematic of the in vivo experiment. (**B**) Motor function was assessed using the Rota-rod test. Mice were pre-trained for 3 days to remain on the rod for 180 sec. Tests were performed 2, 6, 24, and 48 h after last MPTP injection. Values are means ± SEs (*n* = 5–6 mice/group). *** *p* < 0.001 vs. naïve controls and # *p* < 0.05 vs. MPTP controls (the analysis was performed using ANOVA with Fisher’s PLSD procedure).

The observed in vitro neuroprotective and anti-inflammatory effects of EA were confirmed in vivo using an MPTP-induced mouse model of PD. To investigate motor function, mice were pre-trained for 3 days to remain on a rotor rod for 180 s at 30 rpm. They were then orally administered 5 or 80 mg/kg of EA daily for 10 days, and on day 11 intraperitoneally injected 4 times at 2 h intervals to induce a PD-like pathology (Figure 6A). Rota-rod testing showed that all mice in MPTP-treated groups exhibited impaired motor function at 2 h after last MPTP injection, and then progressively recovered slightly. Interestingly, 80 mg/kg EA treatment significantly accelerated motor function recovery (Figure 6B).

### 2.7. EA Diminished MPTP-Induced Dopaminergic Neuronal Death in the Mouse Model

**Figure 7:**
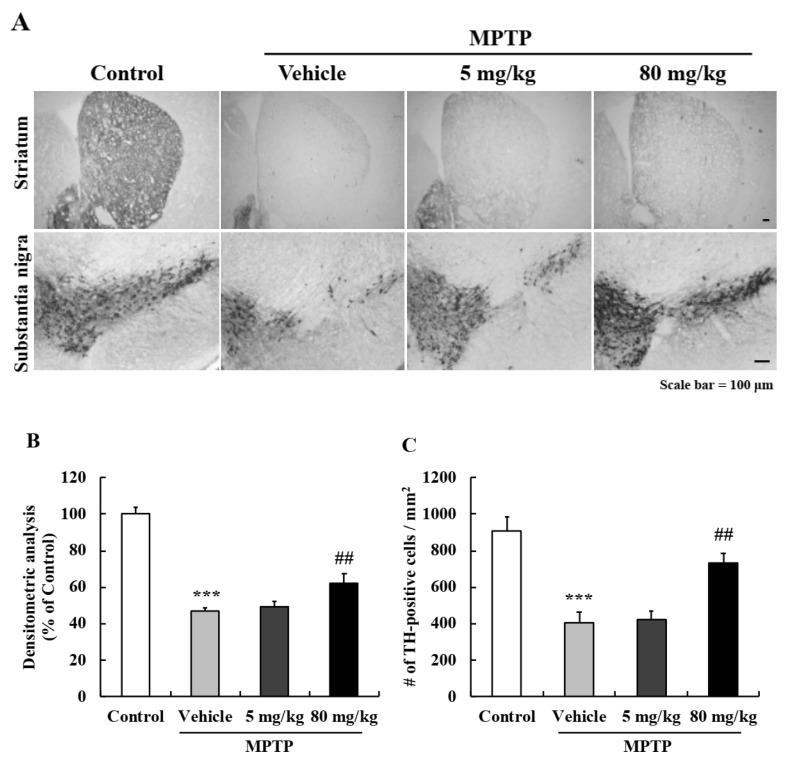
EA prevented dopaminergic neuronal loss in the MPTP-induced murine PD model. (**A**) Immunohistochemistry was used to investigate the neuroprotective effects of EA on the nigrostriatal pathway. Scale bar = 100 µm. (**B**) Densitometric analysis was performed to quantify TH levels in the striatum. (**C**) TH-positive dopaminergic neurons in the substantia nigra were counted, and results are presented as means ± SEs (*n* = 5–6 mice/group). *** *p* < 0.001 vs. naïve controls, ## *p* < 0.01 vs. MPTP controls (the analysis was conducted by ANOVA with Fisher’s PLSD procedure).

The nigrostriatal pathway is a major dopamine pathway that connects substantia nigra (SN) and striatum (STR) and is responsible for motor function. To investigate the neuroprotective effect of EA, we performed immunohistochemistry (IHC) using tyrosine hydroxylase (TH) antibody (a dopamine neuronal marker). Representative images indicated TH levels were significantly lower in all MPTP-treated groups than in vehicle controls, and that 80 mg/kg EA pretreatment significantly attenuated this MPTP-induced dopaminergic neuronal loss in both STR and SN (Figure 7A). TH levels were assessed by densitometry in STR, and TH-positive neurons in SN were counted (Figure 7B,C). Results demonstrated that EA protected against MPTP-induced dopaminergic neuronal damage.

### 2.8. EA Suppressed Astroglial Activation in the PD Mouse Model

**Figure 8:**
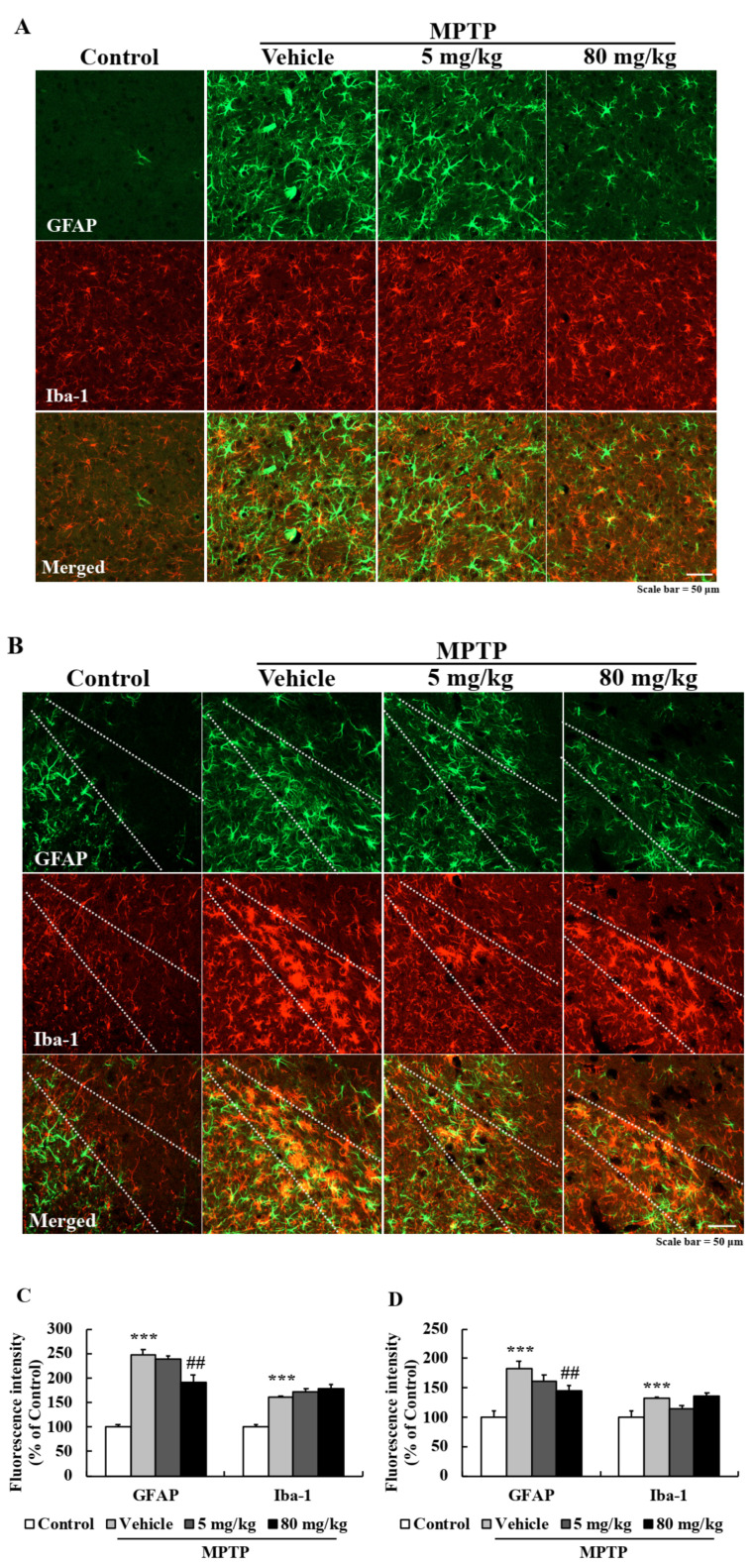
EA reduced MPTP-induced astroglial activation in the nigrostriatal pathway. (**A**,**B**) Striatum (**A**) and substantia nigra (**B**) sections were double immunostained using GFAP (astrocyte marker) and Iba-1 (microglia marker) antibodies. Scale bar = 50 µm. (**C**,**D**) Quantitative analysis of GFAP (green) and Iba-1 (red) fluorescence intensities. Results are presented as means ± SEs (*n* = 5–6 mice/group). *** *p* < 0.001 vs. naïve controls and ## *p* < 0.01 vs. MPTP controls (ANOVA with Fisher’s PLSD procedure).

Chronic neuroinflammation is a characteristic of PD and causes neurodegeneration. We performed double IHC using GFAP and Iba-1 (a known microglia marker) antibodies to determine whether EA pretreatment reduced MPTP-induced glial activation. Interestingly, EA effectively suppressed MPTP-induced astroglial activation in STR and SN (Figure 8A,B) but did not inhibit MPTP-induced microglial activation. Bar graphs of fluorescence intensity also showed EA significantly suppressed astroglial activation (Figure 8C,D). These results showed EA has anti-inflammatory as well as neuroprotective effects.

## 3. Discussion

The etiological causes of most neurodegenerative diseases, including PD, are complicated and have not been fully elucidated, although they are known to involve oxidative stress, mitochondrial dysfunction, and chronic neuroinflammation [5,25,26], which suggests it might be necessary to administer combinations of drugs to treat PD.

Studies on cells, animal models, and postmortem brain tissues indicate dopaminergic neuron apoptosis is a characteristic of PD [27,28,29]. Apoptosis is the major programmed cell death process, and caspase-3 is a key factor in the apoptotic pathway during neurodegeneration [13]. The apoptotic cascade is controlled by the Bcl-2 family proteins, including Bax, Bad, and Bim (pro-apoptotic) and Bcl-2, Bcl-x, and Bcl-w (anti-apoptotic), which regulate mitochondrial permeability [30,31]. Our data showed that Bcl-2/Bax balance was destroyed and caspase-3 was activated by MPP^+^, and that this resulted in the apoptosis of primary cultured neurons, and also that EA pretreatment effectively suppressed MPP^+^-induced reductions in cell viability and disruptions of apoptotic factors (Figure 2).

Mitochondria are important organelles and are responsible for cellular respiration, bioenergetics, and maintaining homeostasis, and mitochondrial dysfunction is a characteristic of PD [26,32]. In particular, the genes mutated in PD include *PINK1*, *PARKIN*, and *LRRK2*, which are related to impaired mitochondrial biogenesis and trafficking, altered mitochondrial dynamics, and ETC dysfunction leading to excessive ROS levels [33]. In the present study, MPP^+^ decreased MMPs by blocking complex I of the ETC, and 100 μM EA suppressed this effect. Seahorse XF Cell Mito Stress testing revealed MPP^+^ damaged mitochondrial respiratory function, and that this too was suppressed by EA by inhibiting proton leakage and preventing MPP^+^-induced reductions in ATP production. Moreover, EA also suppressed the increase in ROS levels resulting from MPP^+^-induced mitochondrial damage, indicating EA also has strong antioxidant activity (Figure 3). Previous in vitro studies have reported that various lichen metabolites have antioxidant effects based on the results of DPPH testing and assessments of free radical scavenging and reducing power [34,35,36]. In addition, the neuroprotective effects of antioxidants in neurodegenerative diseases have been well established [37,38]. However, no study has reported that lichen metabolites improve mitochondrial function. The present study shows for the first time that EA a lichen metabolite improves neuronal mitochondrial function and has antioxidant effect in an in vitro PD model.

Reports indicate a correlation exists between neuroinflammation and PD, and that this neuroinflammation is caused by the upregulations of inflammatory cytokines and the activations of glial cells [39,40,41,42]. Previously, it was reported that the lichen metabolite usnic acid had an anti-inflammatory effect in an MPTP-induced model of PD by inhibiting the NF-κB pathway in astrocytes [19]. Similarly, the current study also demonstrates EA has anti-inflammatory effects on MPP^+^-induced reactive astrocytes, and that it effectively blocked the MPP^+^-induced nuclear translocation of p65 and phosphorylation of IκBα, thus inhibiting the NF-κB signal pathway. Furthermore, the MPP^+^-induced upregulations of inflammatory factors downstream of NF-κB, i.e., COX-2, CCL-2, and pro-inflammatory cytokines, were effectively blocked by EA (Figure 5). Under pathological conditions, reactive glial cells release pro-inflammatory cytokines such as IL-1β, IL-4, IL-6, TNF-α and interferon (IFN)-γ that induce peripheral immune cell infiltration into brain and induce inflammatory response leading to neuronal degeneration [43,44]. Glial cells are activated into either the A1 or A2 types that act in opposing ways, that is, the former causes neurodegeneration whereas the latter has neuroprotective effects [3,45]. The MPP^+^-induced reactive astrocytes observed in the present study were of the A1 type since pro-inflammatory cytokines/chemokine were stimulated in response to MPP^+^. These findings show EA had strong anti-inflammatory effects in our in vitro model by effectively suppressing A1 type astroglial activation.

Regarding the development of therapeutics or health functional foods, oral administration is preferred because it is more economic and convenient than intraperitoneal or intravenous administration. However, it is difficult to predict bioavailability because times required to reach the small intestine depend on gastric emptying time, and drug bioavailabilities and liver metabolism vary [46,47]. In addition, the blood-brain barrier presents a considerable hurdle and is responsible for the poor success rates reported for drugs designed to target the brain [48]. In the present study, to improve bioavailability, EA was administered orally at up to 80 mg/kg, which was enabled by its relatively low toxicity than usnic acid. Motor function impairment and loss of dopamine neurons caused by MPTP were significantly suppressed by EA pretreatment (Figure 6 and Figure 7), and EA also effectively attenuated MPTP-induced astroglial activation in the STR and SN (Figure 8).

In conclusion, we report that EA effectively protected primary neurons and suppressed astroglial activation in in vitro and in vivo models of PD. Furthermore, these neuroprotective effects were found to be mediated by stabilizing neuronal mitochondrial function and ameliorating neuroinflammation-associated A1-astroglial activation. Our findings suggest that EA evokes multiple neuroprotective mechanisms and that EA should be considered an effective therapeutic agent for the prevention and treatment of PD pathologies.

## 4. Materials and Methods

### 4.1. Reagents

MPTP (1-methyl-4-phenyl-1,2,3,6-tetrahydropyridine), MPP^+^ (1-methyl-4-phenylpyridium), MTT (3-[4,5-dimethyl-2-thiazolyl]-2,5-diphenyl-2H-tetrazolium bromide), rotenone, antimycin A, CCCP (carbonyl cyanide 3-chlorophenylhydrazone), oligomycin, and corn oil were obtained from Sigma-Aldrich (St. Louis, MO, USA). Evernic acid (EA) was purchased from Santa Cruz Technology (Santa Cruz, CA, USA). DCF-DA (2′-7′-di-chlorofluorescein diacetate), MitoTracker^TM^ Red CMXRos, Alexa Flour 488, Alexa Flour 568, and DAPI (4’,6-diamidino-2-phenylindole) were from Invitrogen (Carlsbad, CA, USA), and Western blot detection reagent (ECL solution) was purchased from Advansta (San Jose, CA, USA).

### 4.2. High-Performance Liquid Chromatography (HPLC)

Dry lichen extracts were dissolved in 2 mL of acetone and subjected to HPLC (SHIMADZU, LC-20A) using YMC-Pack ODS-A (150 × 3.9 mm I.D.) reverse-phase column fully endcapped C18 material (particle size, 5 µm; pore size, 12 nm). The sample injection volume was 10 µL, and elution was performed at a flow rate of 1 mL/min under the following conditions: column temp, 40 °C; solvent system, methanol: water: phosphoric acid (80:20:1, *v*/*v*/*v*). Eluant was monitored using a photodiode array detector (SPD-M20A) over the wavelength range 190–800 nm. Observed peaks were scanned between 190 and 400 nm. Evernic acid (tR = 4.30 ± 0.2 min) isolated from *Evernia prunastri* was used as a standard.

### 4.3. Primary Neuron Culture

Primary neuron cultures were performed as previously described [49]. Briefly, cortical tissues from embryonic day 18–19 Sprague Dawley rats were excised in ice-cold Hanks’ balanced salt solution (HBSS) buffer (Welgene Inc., Daegu, Korea) containing 0.1 mg/mL gentamycin and incubated for 15 min in 2 mg/mL of trypsin. Tissues were dissociated by trituration, and the cells obtained were plated on poly-l-lysine coated culture dishes in Neurobasal medium (Gibco; Rochester, NY, USA) supplemented with 2% B-27 (Gibco; Rochester, NY, USA), 2 mM L-glutamine, and 25 μM glutamate for 24 h and then transferred to glutamate free Neurobasal medium. Experiments were performed 7 days after plating when most neurons had matured.

### 4.4. MTT Assay

Primary neurons (5 × 10^5^ cells/mL) were seeded in 96-well plates, cultured for 7 days, and pre-treated with several lichen extracts (A2~A48) or EA for 6 h, and then co-treated with MPP^+^ 500 μM for 24 h. Media were then removed, and 200 μL of a 0.5 mg/mL MTT solution in PBS was added to each well. Cells were then incubated at 37 °C for 4 h, MTT solution was removed, and cells were lysed in solubilization solution (DMSO:ethanol = 1:1). Amounts of formazan produced were quantified by measuring absorbance using a Multiskan FC microplate reader at 560 nm (Thermo Fisher Scientific; Waltham, MA, USA).

### 4.5. Mitochondrial Membrane Potential (MMP) Measurement

Primary neurons (2.5 × 10^5^ cells/mL) were seeded in medium in Neurobasal a confocal dish and cultured for 7 days, and pre-treated with 100 μM of EA for 6 h then co-treated with EA and MPP^+^ for 6 h. Cells were then treated with Mitotracker red, which accumulates in an MMP dependent manner in live cells, for 20 min at 37 °C. Representative images were obtained using a ZEISS LSM800 confocal microscope (ZEISS; Oberkochen, Germany).

### 4.6. Measurement of Cellular Mitochondrial Respiration

The Seahorse XF Cell Mito Stress Test (Agilent Technologies; Santa Clara, CA, USA) was performed according to the manufacturer’s instructions. In brief, primary neurons were cultured in XF cell culture microplates, and pre-treated with 100 μM EA for 6 h and co-treated 500 μM MPP^+^ for 6 h. Oligomycin (1 μM), CCCP (1 μM), and rotenone (0.5 μM) plus antimycin A (0.5 μM) were injected sequentially through ports in Seahorse Flux Park Cartridges. Oxygen consumption rates were measured by extracellular flux analyzer, the private assay medium (supplemented with 25 mM D-glucose, 25 mg/L sodium pyruvate, and 500 μM L-glutamine, pH 7.4) for oxygen consumption rate measurement was prepared.

### 4.7. Reactive Oxygen Species (ROS) Assay

Primary neurons (5 × 10^5^ cells/mL) were seeded in black 96-well plates and pre-treated with 0.1, 1, 10, or 100 μM of EA for 6 h and co-treated 500 μM MPP^+^ at 6 h. Media were then removed, and cells were incubated with 80 μM DCF-DA in neurobasal at 37 °C for 30 min and then washed twice with PBS. Fluorescence intensities were measured at 10 min intervals after EA+MPP^+^ treatment using a Glomax fluorescence plate reader (Promega Corportaion; Madison, WI, USA).

### 4.8. Western Blot Analysis

After treatments, cells were subjected to SDS-PAGE (sodium dodecyl sulfate-polyacrylamide gel electrophoresis), and protein concentrations in supernatants were measured using a Bio-Rad (Hercules, CA, USA) protein assay kit and bovine serum albumin (BSA) as the standard. Proteins in supernatants (20 μg protein per lane) were then separated by 10% SDS-PAGE and transferred to Immobilon-P^SQ^ membranes (Millipore; Burlington, MA, USA). Membranes were immediately placed in 5% skim milk for 30 min and then incubated with the following primary antibodies; cleaved caspase 3 (rabbit polyclonal; Cell Signaling, Danvers, MA, USA), caspase 3, Bax, t-IκBα (rabbit polyclonal; Santa Cruz Biotechnology, Santa Cruz, CA, USA), Bcl-2, *p*-IκBα, COX-2 (mouse monoclonal; Santa Cruz Biotechnology, Santa Cruz, CA, USA), GFAP (rabbit polyclonal; Abcam, MA, USA), and β-actin (mouse monoclonal; Sigma-Aldrich, St. Louis, MO, USA) in Tris-HCl-based buffer containing 0.2% Tween 20 (TBS-T; pH 7.5) overnight at 4 °C. Membranes were then washed and incubated with secondary monoclonal anti-mouse and polyclonal anti-rabbit antibodies (1:10,000; Santa Cruz Biotechnology, Santa Cruz, CA, USA) in TBS-T for 2 h. Horseradish peroxidase conjugated secondary antibody labeling was detected by enhanced chemiluminescence (ECL) using a cooled CCD camera system (ATTO Ez-Capture II; Atto Corp., Tokyo, Japan). Relative protein levels were quantified by densitometry using β-actin as the standard.

### 4.9. Primary Astrocyte Culture

Primary astrocytes were isolated from Sprague Dawley rat pups at postnatal day (PND) 1 or 2 (Daehan Biolink Co. Ltd., Chungbuk, South Korea). Briefly, cortices were dissected and diffused in ice-cold HBSS buffer (Welgene Inc., Daegu, South Korea). Cells were then treated with 0.25% trypsin for 30 min at 37 °C, washed with HBSS, mechanically dissociated, and plated in Dulbecco’s modified Eagle’s medium/nutrient mixture F-12 (DMEM/F12 medium containing 10% FBS on poly-L-lysine coated plates. Cultures were maintained in the same solution and used for experiments after 14–18 days in vitro.

### 4.10. Immunocytochemistry

Primary astrocytes were seeded in 60-mm poly-L-lysine-coated plastic culture dishes, pre-treated with 100 μM EA for 6 h, and then treated with 500 μM MPP^+^ for 24 h, washed with PBS, fixed with 4% PFA in PBS (pH 7.4) for 20 min at 37 °C, and blocked with TBS-TS (0.1% Triton X-100/3% goat serum) for 30 min at room temperature. Cells were then incubated with primary antibodies for GFAP (mouse monoclonal; Cell Signaling, Danvers, MA, USA) or p65 (rabbit monoclonal; Cell Signaling, Danvers, MA, USA) at 4 °C overnight, washed with TBS-TS and then TBS, incubated with anti-mouse IgG labeled with Alexa Fluor 488 and anti-rabbit IgG labeled with Alexa Fluor 568 (Invitrogen; Carlsbad, CA, USA) for 3 h at room temperature, and washed with TBS. Cells were then treated with DAPI (1 μg/mL) in TBS for 20 min to stain nuclei, and images were acquired using a ZEISS LSM800 confocal microscope (ZEISS; Oberkochen, Germany).

### 4.11. RNA Isolation and Real-Time Polymerase Chain Reaction (Real-Time PCR)

Cells were homogenized with Trizol reagent (Invitrogen; Carlsbad, CA, USA), chloroform was added, and shaken vigorously for 15 min. The aqueous phase was then transferred to fresh tubes, isopropanol was added, incubated for 15 min at 4 °C, and centrifuged for 15 min at 12,000 *g*. Supernatants were removed and pellets were washed with 75% ethanol and centrifuged for 5 min at 8000 *g*. The RNA pellets obtained were dried and dissolved in diethyl pyrocarbonate water, and mRNA concentrations were calculated. mRNA was reverse transcribed to cDNA using SuPrimeCript RT Premix (Genetbio Inc.; Daejeon, South Korea). Real-time PCR analysis was performed using SYBR green master mix (BIOLINE; Taunton, MA, USA) and the CFX Connect System (Bio-rad Inc.; Hercules, CA, USA). Primer sequences were as follows; IL-1β (5′-AAA ATG CCT CGT GCT GTC TG-3′ and 5′-CCA CAG GGA TTT TGT CGT TG-3′); IL-6 (5′-TCA TTC TGT CTC GAG CCC AC-3′ and 5′-GAA GTA GGG AAG GCA GTG GC-3′); CCL2 (5′-AGC ATC CAC GTG CTG TCT C-3′ and 5′-GAT CAT CTT GCC AGT GAA TGA G-3′); TNF-α (5′-ATT GCT CTG TGA GGC GAC TG-3′ and 5′-GGG GCT CTG AGG AGT AGA CG-3′); GAPDH (5′-AGA CAG CCC CAT CTT CTT GT-3′ and 5′-ACG GTG AGT CTT CTG ACA CC-3′).

### 4.12. Animals and Treatments

Male C57BL/6 mice (6 weeks old, 20–23 g) were obtained from Daehan Biolink Co. Ltd. (Chungbuk, South Korea). Animals were maintained under temperature- and light-controlled conditions (20–23 °C under a 12 h light cycle) and provided food and water *ad-lib*. Animals were randomly allocated to four groups of 10, that is, to a vehicle control group, an MPTP control group, an MPTP + 5 mg/kg EA group, or an MPTP + 80 mg/kg EA group. All animals were acclimatized for 1 week prior to drug administration. EA (5 or 80 mg/kg dissolved in corn oil containing 5% DMSO) was administered to mice daily (oral injection) for 10 days and then on day 11, MPTP was injected intraperitoneally at 20 mg/kg four times at 2 h intervals. Two independent in vivo experiments were performed to verify the reproducibility of results. A schematic of the in vivo experiments is provided in Figure 6. The animal protocol used in this study was reviewed and approved beforehand by the Pusan National University Institutional Animal Care Committee (PNU-IACUC; Approval Number PNU-2020–2491).

### 4.13. Motor Performance Testing

Motor performance was assessed using a Rota-rod apparatus, as previously described [50]. All mice were pre-trained for 3 days to maintain themselves on the rod for 180 s. Training sessions involved four consecutive runs at a rod speed of 30 rpm. All mice were tested 2, 6, 24, 48, and 72 h after final MPTP administration. On days following the completion of motor performance testing, mice were sacrificed for histological analyses.

### 4.14. Tissue Preparation

For histological studies, mice were anesthetized with diethyl ether and perfused intracardially with 0.9% NaCl in 0.1 M PBS (pH 7.4). After fixation with 4% paraformaldehyde (PFA) in 0.1 M PBS, brains were removed, placed in the same fixative solution at 4 °C overnight, and then transferred to 30% sucrose. Cryoprotected brains were serially sectioned at 40 μm in the coronal plane using a freezing microtome (MICROM; Walldorf, Germany) and stored at 4 °C in Dulbecco’s phosphate-buffered saline (DPBS) solution containing 0.1% sodium azide.

### 4.15. Diaminobenzidine (DAB) Immunohistochemistry

Briefly, to block endogenous peroxidase activity, brain sections were treated with 0.6% H_2_O_2_ in Tris-buffered saline (TBS; pH 7.5), blocked in TBS-TS for 30 min, and incubated with primary anti-TH antibody (mouse monoclonal; Chemicon, Temecula, CA, USA) in TBS-TS at 4 °C. Sections were further processed using appropriate biotinylated secondary goat anti-mouse IgG antibodies (Vector Laboratories; Burlingame, CA, USA) at room temperature for 3 h, incubated in ABC solution (ABC reagent Elite Kit, Vector Laboratories; Burlingame, CA, USA) at room temperature for 1 h, and developed with diaminobenzidine (DAB) solution. Images were obtained using a Nikon ECLIPSE TE 2000-U microscope (Nikon, Tokyo, Japan). Densitometric analysis in STR was performed using FluorChem SP software (Alpha Innotech, San Leandro, CA, USA) and TH+ neurons were counted in entire extents of the SN by the same blinded investigator.

### 4.16. Double Fluorescence Immunohistochemistry

Brain sections were blocked with TBS-TS for 30 min and incubated with primary antibodies (anti-GFAP mouse monoclonal (Cell Signaling, Danvers, MA, USA) and anti-Iba-1 rabbit polyclonal (Wako, Tokyo, Japan)) in TBS-TS overnight at 4 °C. They were then incubated with secondary anti-mouse IgG labeled with Alexa Fluor 488 and anti-rabbit IgG labeled with Alexa Fluor 568 for 3 h at room temperature, washed with TBS, and mounted onto slides using aqueous and dry mounting medium (Biomeda Corp.; Foster City, CA, USA). Images were acquired using a ZEISS LSM800 confocal microscope (ZEISS; Oberkochen, Germany).

### 4.17. Statistical Analysis

The significances of intergroup differences were determined by analysis of variance (ANOVA) with Fisher’s protected least significant difference (PLSD) procedure. *p* values of <0.05 were considered statistically significant. The analysis was performed using Statview software (Version 5.0.1., SAS Institute Inc.; Cary, NC, USA).

## Data Availability

The data that support the findings of this study are available from the corresponding author upon reasonable request.
